# Interdisciplinary Integrative Oncology Group-Based Program: Evaluation of Long-Term Effects on Resilience and Use of Complementary and Alternative Medicine in Patients With Cancer

**DOI:** 10.1177/15347354241269931

**Published:** 2024-08-18

**Authors:** Marek Jonas Von Reusner, Bettina Märtens, Stephanie Barthel, Axel Weiser, Yvonne Ziert, Diana Steinmann, Burcu Babadağ-Savaş

**Affiliations:** 1 Department of Radiotherapy, Hannover Medical School, Hannover, Germany; 2 CCC Hannover (Claudia von Schilling Center), Klaus Bahlsen Center for Integrative Oncology, Hannover Medical School, Hannover, Germany; 3 Quality Management, Hannover Medical School, Hannover, Germany; 4 Institute of Biometrics, Hannover Medical School, Hannover, Germany

**Keywords:** long-term effects, Complementary and Alternative Medicine, CAM, integrative, oncology, resilience, quality of life, naturopathy, group program, care

## Abstract

**Background:**

Cancer often causes reduced resilience, quality of life (QoL) and poorer overall well-being. To mitigate these problems, complementary and alternative medicine (CAM) is widely used among patients with cancer. This study aimed to evaluate the long-term effects of an interdisciplinary integrative oncology group-based program (IO-GP) on the resilience and use of CAM in patients with cancer.

**Methods:**

This was a prospective, observational, single-center study. Resilience (RS-13), CAM usage (I-CAM-G), QoL (SF-12) and health-related lifestyle factor (nutrition, smoking, alcohol consumption and physical exercise) data were collected for 70 patients who participated in a 10-week IO-GP between January 2019 and June 2022 due to cancer. The IO-GP is offered at the setting of a university hospital and is open to adult patients with cancer. It contains elements from mind-body medicine and positive psychology, as well as recommendations on healthy diet, exercise and CAM approaches. Patients who completed the IO-GP at least 12 months prior (1-4.5 years ago) were included in this study. Statistical analysis included descriptive analysis and parametric and nonparametric tests to identify significant differences (*P* < .05).

**Results:**

Resilience increased significantly ≥12 months after participation in the IO-GP (n = 44, *P* = .006, *F* = 8.274) and had a medium effect size (*r* = .410). The time since the IO-GP was completed (“12-24 months,” “24-36 months,” and “>36 months”) showed no statistically significant interaction with changes in resilience (*P* = .226, *F* = 1.544). The most frequently used CAM modalities within the past 12 months were vitamins/minerals (85.7%), relaxation techniques (54.3%), herbs and plant medicine (41.1%), yoga (41.4%) and meditation (41.4%). The IO-GP was the most common source informing study participants about relaxation techniques (n = 24, 64.9%), meditation (n = 21, 72.4%) and taking vitamin D (n = 16, 40.0%). Significantly greater levels of resilience were found in those practicing meditation (*P* = .010, *d* = −.642) or visualization (*P* = .003, *d* = −.805) compared to non-practitioners.

**Conclusion:**

IO-GPs have the potential to empower patients with cancer to continue using CAM practices—especially from mind-body medicine—even 1 to 4.5 years after completing the program. Additionally, resilience levels increased. These findings provide notable insight into the long-term effects of integrative oncology interventions on resilience and the use of CAM, especially in patients with breast cancer.

## Introduction

In recent decades, cancer diagnoses have increased worldwide.^1^ Being diagnosed with cancer poses significant challenges for individuals affected by it, often resulting in physical and psychological burdens,^2,3^ related both to cancer itself (anxiety, depression, and stress),^4^ and to treatment-related symptoms (menopausal symptoms, pain, fatigue, and sleep disturbance).^5^ These burdens can in turn lead to increased distress and, consequently, decreased quality of life (QoL) and resilience.^5,6^ To navigate cancer-related physical, psychological and emotional challenges, resilience represents a meaningful protective factor for people with cancer.^7,8^ By definition, resilience is the ability to adapt to stressors and to maintain and regain mental health during challenging life experiences.^9^ However, resilience is a dynamic process that can change and evolve over time.^10^

In this context, existing literature shows that complementary and alternative medicine (CAM) approaches can play a significant supportive role in promoting resilience in patients with cancer.^9,11^ CAM, on its own or when included in clinical practice as “integrative medicine,” encompasses a diverse range of health care measures and treatments, such as nutritional approaches, herbal remedies, dietary supplements, acupuncture, homeopathy, and psychological techniques from mind-body medicine, such as mindfulness, breathing exercises, relaxation methods, yoga, meditation, visualization, and stress management.^12^

The use of CAM is widespread among patients with cancer and generally increases after receiving a cancer diagnosis.^13–16^ Commonly reported reasons for the utilization of CAM by patients with cancer include enhancing QoL, general well-being and the ability to relax, to support the body in fighting cancer, and to alleviate cancer-related long-term burdens, while the use of CAM generally results in high satisfaction and low rates of unwanted side effects.^14,17^

In that regard, it is worth noting that the German Working Group for Gynecological Oncology (AGO) and the S3 guidelines for complementary medicine in oncological treatment settings recommend approaches such as Mindfulness-Based Stress Reduction (MBSR), meditation, physical exercise/sport, and yoga to patients with gynecological cancer as complementary treatments with a high level of evidence.^18,19^

Participating in an interdisciplinary integrative oncology group-based program (IO-GP) can play a major role for integrating CAM approaches alongside conventional treatments for patients with cancer. In Germany, integrative oncological, multimodal treatment concepts have been established in the form of day-units, for example, in “Evangelische Kliniken Essen-Mitte”^20,21^ and “Immanuel Hospital Berlin,”^22,23^ while studies on these integrative programs have demonstrated their effectiveness in promoting QoL and mental health and alleviating fatigue, anxiety, and depression.^21,23^ Furthermore, it turned out to be of great relevance that these programs are conducted under guidance of an interdisciplinary expert team of integrative oncology specialists.^24,25^ Additionally, multimodal treatment concepts and group interventions have been associated with enhancements in resilience,^9,11,25^ the adoption of healthy lifestyle behaviors,^26^ and the reduction of cancer-related symptoms.^4,27^

Indeed, it is important to note that studies primarily focus on short-term effects of group-based interventions, with limited follow-up investigations typically lasting up to 6 months. In that regard, previous studies have shown improvements in QoL^23^ and healthy lifestyle behaviors^26^ during 6-month follow-up periods. These findings suggest that IO-GPs may have sustained effects on patients with cancer.

To gain further knowledge about the potential lasting impact of IO-GPs, particularly several years after their completion, this study aimed to investigate the long-term effects of an IO-GP in our university hospital, focusing on resilience and the utilization of CAM 1 to 4.5 years after participation. Thus, the objectives of this study were as follows:

Primary objective:

- Evaluation of the long-term effects of the interdisciplinary integrative oncology group-based program on changes in resilience.

Secondary objectives:

- Evaluation of the usage behavior of CAM after participation in the group-based program.
- Investigation of connections between resilience, the usage behavior of CAM, quality of life and lifestyle factors.

## Materials and Methods

### Study Design

This was a prospective, observational, single-center study with the aim of evaluating the long-term effects of a 10-week IO-GP carried out at the Department of Radiotherapy of the Hannover Medical School (MHH) in Germany.

### Participants

**Figure 1.**
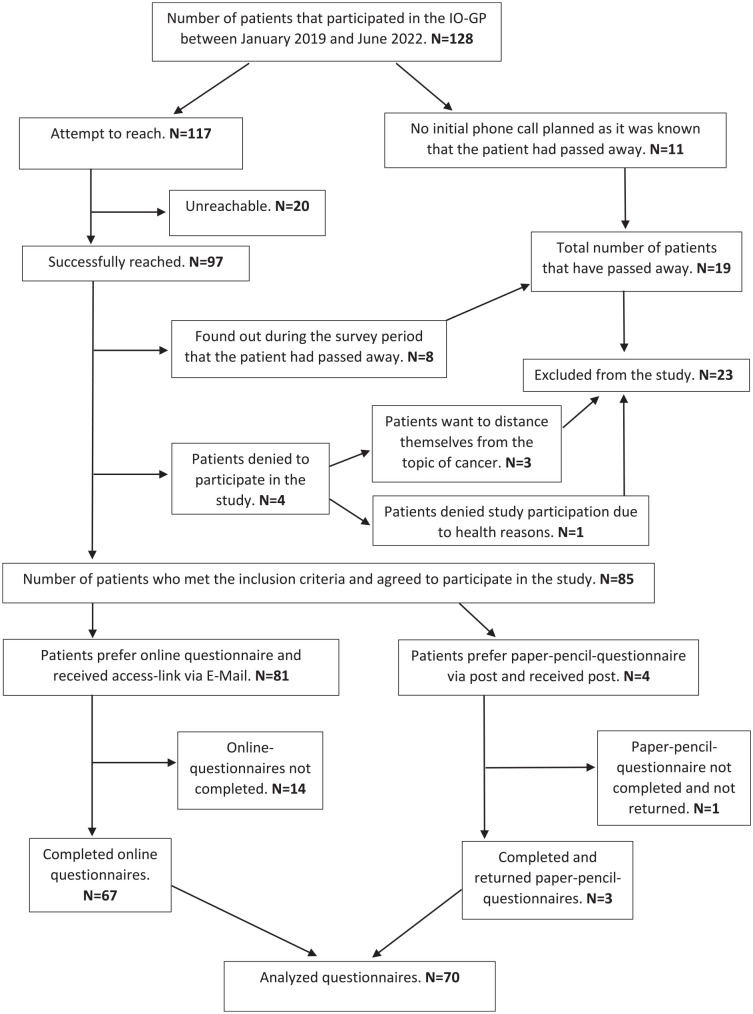
Flow chart of the study participants.

All patients who participated in the IO-GP between January 2019 and June 2022 (≥12 months ago; range: 1-4.5 years ago) in the context of cancer were contacted. The data were collected in July and August 2023. In this context, the flow chart (Figure 1) demonstrates that a total of 128 patients participated in the IO-GPs between January 2019 and June 2022. Knowing that 11 patients had passed away, 117 patients qualified to be contacted by telephone, asking for participation, providing information about the study, and ensuring their suitability concerning the inclusion and exclusion criteria. Of the 97 successfully contacted patients, it was found that 8 patients had passed away, so they were excluded from the study. Four patients denied study participation via the personal phone calls, with 3 patients preferring to stay away from the topic of cancer and 1 patient not wanting to participate due to health reasons. Consequently, 85 agreed to participate in the study and likewise met the inclusion criteria. Ultimately, 70 patients completed the questionnaires.

The inclusion criteria were (1) having attended at least 1 course of the IO-GP between January 2019 and June 2022, (2) being over 18 years of age and (3) providing consent to participate in the study. Patients with significant physical or psychological limitations that did not guarantee reliable answers to the questionnaire were excluded from the study.

### Interdisciplinary Integrative Oncology Group-Based Program

In the Department of Radiotherapy at the Hannover Medical School, an IO-GP was established in October 2018 for patients with cancer. Since January 2022, this program has been provided by the Klaus-Bahlsen Center for Integrative Oncology within the framework Comprehensive Cancer Center Hannover (CCC-H). The IO-GP is exclusively financially supported by the foundations Rut- und Klaus-Bahlsen-Stiftung and Förderstiftung MHH plus. There was no financial support by insurance companies. IO-GPs are still ongoing and are open for all interested, adult patients currently undergoing or have previously received cancer therapy. Participation is free of charge for patients with cancer and independent of the type of cancer. Patients at the university hospital, but also patients who are being treated in other clinics or outpatient institutions, are allowed to take part in the IO-GP. Before participating in the IO-GP, patients receive complementary medical consultations from a qualified doctor. Patient recruitment for the IO-GP is conducted through various channels, such as posters, brochures, business cards, websites,^28^ and naturopathic consultations led by physicians and nurses. Interested patients can contact the center by email or telephone to schedule a first appointment in which they receive initial information on integrative oncology approaches.

**Figure 2.**
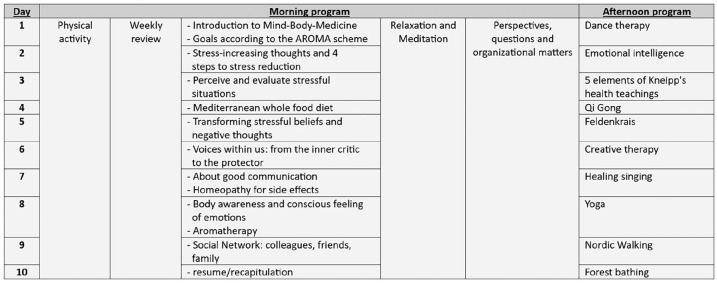
Exemplary schedule of the interdisciplinary integrative oncology group-based program.

The timeframe of the IO-GP consists of a total of 50 hours. Over a period of 10 weeks, there was a 5-hour course 1 day per week, resulting in 10 courses in total. Each 10-week group has an average of 12 participants and is led by an interdisciplinary team comprising physicians, psychologists, nurses, physiotherapists, social scientists, and specialists in various therapeutic modalities, such as yoga, music, and dance. An important emphasis of the IO-GP is aligned with elements from mind-body medicine, such as meditation, relaxation techniques and dealing with negative thoughts.^25,28^ A typical schedule^29^ of the 10-week program is shown in Figure 2.

### Measurement and Assessment

The data were collected at least 12 months after the patients had completed the IO-GP, either by the online survey platform “SoSci Survey” or by paper-pencil version using the questionnaires detailed below.

### Sociodemographic Data and Disease-, Treatment-, and Health-Related Factors

Information on sociodemographic data and disease- and treatment-related factors was collected by the researcher according to a prior study on the IO-GP^25^ and consisted of 7 items on sociodemographic characteristics (eg, age, gender, etc.), questions about medical records (diagnosis, current treatments, completed treatments, cancer recurrence, and further diseases) and data on the IO-GP (number of completed courses). Furthermore, health-related information was gathered with 10 items relating to patients’ lifestyle behaviors, including questions on “nutrition,” “smoking,” “alcohol consumption,” and “exercise.” Nutritional questions on vegetable and fruit consumption are based on the recommendations of the German Nutrition Society,^30^ and questions on “olive oil consumption,” “red meat, sausage and fast food,” and “sweets and sugar” are adjusted on the German Mediterranean Diet Adherence Screener (MEDAS).^31^ Questions on nutrition were developed in collaboration with a nutritionist from the interdisciplinary integrative oncology expert team. Information on smoking, alcohol consumption and physical activity is oriented toward the general health-related lifestyle recommendations of the World Health Organization (WHO).^32,33^

### Resilience Scale (RS-13)

Resilience was measured using the German Resilience Scale (RS-13), which comprises 13 items. Each item was rated on a scale from 1 to 7, where higher scores indicate greater resilience. The total resilience score results from summing the scores of all 13 items. A total score of 13 to 66 is associated with low resilience, scores of 67 to 72 with moderate resilience and scores of 73 to 91 with high resilience. The internal consistency of the RS-13 scale was α = .90.^34^

### Standardized International Complementary and Alternative Medicine Questionnaire

To assess the prevalence of CAM usage within the past 12 months, the German questionnaire “I-CAM-G” by Lo Re et al,^35^ which is based on the standardized International Complementary and Alternative Medicine Questionnaire (I-CAM-Q) of Quandt et al,^36^ was used in this study.

The I-CAM-G consists of 4 sections: (1) visited health care providers offering CAM, (2) CAM treatments received by a physician, (3) the use of herbal drugs and dietary supplements, and (4) self-help practices. In all 4 sections, the I-CAM-G asks further questions on the quantity of CAM modalities used, the reason for CAM usage and how helpful it was. With the authors’ permission,^35^ modifications were made to the 4 sections of the I-CAM-G, specifically aligning them with the objectives of this study. Consequently, participants were asked about the quantity of CAM used, whether they used these methods in relation to their cancer disease, whether they became aware of the CAM modality through the IO-GP, how helpful it was and whether they had used the CAM modality before participating in the IO-GP.

### General Health Questionnaire (SF-12)

Health-related QoL was evaluated with the General Health Questionnaire (SF-12), which is based on the SF-36.^37^ Specifically, the German self-report version of the standardized SF-12v2 questionnaire,^38^ which includes a physical and a mental scale, was utilized. The questionnaire contains 12 questions and encompasses 8 main dimensions: general health, physical functioning, role function and bodily pain, making up the physical scale (SF-P), while emotional role function, psychological wellbeing, negative emotions and social functioning pertain to the mental health scale (SF-M). The subscales of the German SF-12 show an internal consistency of α > .70.^38^

For evaluation, the respective weighting factors are assigned to the study participants’ answers. Summing the weighting factors with a corresponding constant consequently results in a score for the physical scale and another score for the mental scale. The average physical and mental QoL scores were both individually normed at 50, with a standard deviation (SD) of 10, while a score above 50 indicates a higher QoL in the respective dimension.^38^

### Sample Size Calculation

Regarding the primary study objective (changes in resilience), sample size planning was based on a previous study on the IO-GP^25^ and it was calculated that 261 patients would be required to detect an effect of a change in the resilience of the MD of 2.7, with a SD of 15.5, a power of 80% and a significance of *P* < .05. At the time of the survey, baseline resilience values were available for approximately 80 patients, also encompassing participants from the previous study.^25^ Consequently, the sample size for the primary study objective was n = 80, and ultimately, out of the 80 patients, 44 participated in the follow-up for resilience changes.

### Statistical Analysis and Data Distribution

Statistical analyses were performed by using IBM SPSS Statistics (Statistical Package for Social Sciences Version 28.0.1.1.). The descriptive data included sociodemographic data, disease- and treatment-related factors, lifestyle factors and the use of CAM. Stated percentages are related to the valid number of participants in the descriptive statistics. For the resilience (RS-13) and QoL (SF-12) scales, the data distributions were first tested with the Kolmogorov–Smirnov test. Subsequently, the data distribution was evaluated graphically by distribution histograms. The resilience values were determined to be normally distributed, so the means and standard deviations (M ± SD) and parametric tests (ANOVA and Student’s *t* test) were used. The physical (SF-P) and mental (SF-M) QoL values were not normally distributed, so medians and ranges (Mdn; *R*) and nonparametric tests (Mann–Whitney *U* tests) were used. For calculating effect sizes for mean score differences, Cohen’s *d* was used for t tests (defined as |*d*| ≥ .2 small effect, |*d*| ≥ .5 medium effect, and |*d*| ≥ .8 large effect), and *r* was used for ANOVA and the Mann–Whitney *U* test (defined as .1 ≤ *r* < .3 small effect, .3 ≤ *r* < .5 medium effect, and *r* > 5 large effect).^39^ Investigations of changes in resilience from baseline to ≥12 months after participation in the IO-GP were carried out using analysis of variance (ANOVA) with repeated measures and the between-subjects factor “time since the IO-GP was completed” (“12-24 months,” “24-36 months,” and “>36 months”) as a controlling factor.

Missing item values in the current RS-13 were substituted by the corresponding item values from baseline, and in turn, missing item values from baseline in the RS-13 were replaced by the current item values. Regarding missing data in the I-CAM-Q and SF-12, no imputation was performed. Study participants who provided missing values in the SF-12 were not included in the analysis of QoL. Answers in free text fields were checked for suitability (eg, information on “herbal drugs and dietary supplements” was assigned to the correct categories or removed if they were inapplicable). *P* values <.05 were considered to indicate statistical significance. SPSS, by default, conducts analyses by dropping cases for which there are missing values, so the sample sizes may differ in the statistical analyses.

### Ethical Considerations

The study project was approved by the ethics committee of the university (approval number: 10621_BO_K_2022). A declaration of consent and confirmation of the data policy were obtained via the SoSci Survey platform (option in) from patients who completed the online questionnaire, while patients participating in the paper-pencil format provided written consent.

## Results

### Sociodemographic Data and Disease-, Treatment- and Health-Related Factors

Patient characteristics are shown in Table 1. The study population (N = 70) had an average age of 60 ± 11.42 years, and all participants were female (100.0%). Most of the patients were married (67.6%), had high educational levels (56.5%) and were pensioners (46.4%). The majority (68.5%) took part in 9 or 10 courses of the completed 10-week IO-GP. Among the participants, breast cancer was the most frequently reported cancer type (83.1%). Additionally, some patients (40.0%) reported suffering from another illness or serious life situation (eg, cardiovascular disease, GI symptoms, osteoporosis, arthrosis, other cancer diseases, depression, ailments of a relative, or other social issues). Descriptive data on health-related lifestyle factors illustrated that the study population generally showed healthy nutritional behaviors related to the recommendations of the DGE and WHO. Most participants consumed ≥2 portions of fruits, <1 portion of red meat, sausage and fast food, and <1 portion of sweets and sugar per day. Vegetable (≥3 portions) and olive oil consumption (≥4 tablespoons) mostly contrasted with nutritional recommendations. In addition, it is noteworthy that almost all participants (96.9%) were nonsmokers (“0 cigarettes per day”) and stated that they drank “no alcohol” (28.6%) or only “sometimes” (54.3%). Concerning physical exercise, most participants exercised 1 to 2 times per week (47.1%).

**Table 1.** Patient Characteristics.

Sociodemographic data and disease-, and treatment-related factors	M ± SD	
Age (years)	60 ± 11.42	
	n	%
Gender	70	100.0
Female	70	100.0
Male	0	0.0
Marital status	68	100.0
Married	46	67.6
In a relationship	10	14.7
Single	8	11.8
Widowed	4	5.9
Educational level	69	100.0
High school	39	56.5
Intermediate school	14	20.3
Advanced technical certificate	9	13.0
Lower secondary school	4	5.8
Other	3	4.3
Employment	69	100.0
Pensioner	32	46.4
Working	26	37.7
Housemaker	1	1.4
Other	10	14.5
Number of visited courses of the group program	70	100.0
10	22	31.4
9	26	37.1
8	8	11.4
7	6	8.6
<7	5	7.1
“Don’t know”	3	4.3
Cancer diagnosis	65	100.0
Breast cancer	54	83.1
Gynecologic cancer (ovarian, uterus, cervix)	3	4.6
Other cancer^a^	8	12.3
Treatments previously received for the cancer associated with IO-GP participation	69	100.0
No	2	2.9
Yes^b^	67	97.1
Surgery	56	81.2
Radiation	54	78.3
Chemotherapy	37	53.6
Hormone therapy	27	39.1
Other treatments^c^	7	10.1
In current cancer treatment	58	100.0
No	39	67.2
Yes^b^	19	32.8
Hormone therapy	12	20.7
Chemotherapy	3	5.2
Surgery	1	1.7
Radiation	1	1.7
Other treatments^c^	7	12.1
Experienced cancer recurrence	68	100.0
Yes	10	14.7
No	58	85.3
Health-related lifestyle factors	n	%
Vegetables (per day)	69	100.0
≥3 Portions	32	46.4
<3 Portions	37	53.6
Fruits (per day)	70	100.0
≥2 Portions	53	75.7
<2 Portions	17	24.3
Olive oil (per day)	70	100.0
≥4 Tablespoons	11	15.7
<4 Tablespoons	59	84.3
Red meat, sausage and fast food (per day)	69	100.0
≥1 Portion	8	11.6
<1 Portion	61	88.4
Sweets and sugar (per day)	69	100.0
≥1 Portion	20	29.0
<1 Portion	49	71.0
Number of smoked cigarettes (per day)	65	100.0
0	63	96.9
1-10	1	1.5
11-20	1	1.5
Alcohol consumption	70	100.0
Not at all	20	28.6
Sometimes	38	54.3
Multipe times a week	10	14.3
Daily	2	2.9
Physical activity	70	100.0
Not at all	7	10.0
1-2×/week	33	47.1
3-4×/week	22	31.4
>4×/week	8	11.4

Abbreviations: n, number of patients; M, mean; SD, standard deviation.

a Skin cancer, sarcomas, lymphomas, neuroendocrine cancer, lung cancer, CUP.

b More than 1 option could be selected by the patient.

c Immunotherapy/monoclonal antibody therapy, plant-therapy, DIEP flap, rehabilitation, after-care.

### Changes in Resilience From Baseline to ≥12 Months After Completing the IO-GP

A comparison of the mean resilience scores before (baseline) participation in the IO-GP and ≥12 months (range: 1-4.5 years) after participation was conducted (n = 44) and is shown in Table 2. A statistically significant increase in resilience was observed ≥12 months after the IO-GP (baseline: 62.20 ± 13.86 [low resilience] vs ≥12 months: 68.73 ± 9.72 [moderate resilience], *P* = .006, *F* = 8.274), and these changes had a medium effect size (*r* = .410). However, the controlling factor “time since the IO-GP was completed” (“12-24 months,” “24-36 months,” and “>36 months”) showed no statistically significant interaction with changes in resilience (*P* = .226, *F* = 1.544).

**Table 2.** Changes in Resilience From “Baseline” to “≥12 Months” After Completing the IO-GP.

Resilience baseline		Resilience ≥12 months		Resilience change			Resilience change × “Time since the IO-GP was completed” interaction	
n	M ± SD	n	M ± SD	*F*-value *	*P*-value*	Effect size *r*	*F*-value*	*P*-value*
44	62.20 ± 13.86	44	68.73 ± 9.72	8.274	.**006**	.410	1.544	.226

Abbreviations: n, numbers of patients; M, mean; SD, standard deviation.

* Analysis of variance with repeated measures and between-subjects factor; bold *P*-values indicate significant differences (*P* < .05).

Furthermore, the categorical classification of resilience showed the following results: at baseline, 61.4% of the participants displayed low resilience, 20.5% exhibited moderate resilience, and 18.2% demonstrated high resilience. In contrast, at the time of data collection (≥12 months), 38.6% of the respondents exhibited low resilience, 25.0% exhibited moderate resilience, and 36.4% exhibited high resilience. Consequently, the number of patients with “low resilience” decreased in the period after the group program, and the number of patients with “moderate resilience” and “high resilience” increased.

### Use of CAM After Participation in the IO-GP (I-CAM-G)

Table 3 presents descriptive data on the usage of CAM that had been used within the last 12 months. Among the study participants (N = 70), the most frequently visited CAM providers were “other CAM providers” (20.0%), “osteopaths” (18.6%), and “physicians for CAM” (18.6%). Herbal medicine (34.3%), manual therapy (28.6%), and acupuncture (17.1%) were the most common CAM treatments. Moreover, the “herbal medicine and dietary supplements” section revealed that the majority of the patients reported a high usage of vitamins and minerals (85.7%), while vitamin D (57.1%), magnesium (30.0%) and multinutritional supplements (24.3%) were the most common substances. Some patients (41.4%) used “herbs and plant medicine,” mostly mentioning turmeric (12.9%) and herbal teas (7.1%). Furthermore, 28.6% of the respondents stated that they used “homeopathic remedies,” whereas omega-3 oils (18.6%) were mostly reported in the category “other natural drugs.” Finally, regarding “self-help strategies,” relaxation techniques (54.3%), meditation (41.4%), and yoga (41.4%) were practiced the most.

**Table 3.** I-CAM-G Results of All Study Participants.

Number of patients who used this CAM modality within the last 12 months^a^		Did you use this CAM modality in context with your cancer disease?^b^			Did you become aware of this CAM modality through the group program?^b^			How helpful did you find this CAM modality?^b^					Did you already use this CAM modality before participating in the group program?^b^		
	n (%)	n	Yes, n (%)	No, n (%)	n	Yes, n (%)	No, n (%)	n	Very helpful, n (%)	Somewhat helpful, n (%)	Not helpful, n (%)	Don’t know, n (%)	n	Yes, n (%)	No, n (%)
Section 1: “Visited CAM provider (physician or non-physician)”															
Homeopath	8 (11.4)	8	2 (25.0)	6 (75.0)	8	4 (50.0)	4 (50.0)	7	4 (57.1)	3 (42.9)	0	0	7	3 (42.9)	4 (57.1)
Acupuncturist	12 (17.1)	12	6 (50.0)	6 (50.0)	12	3 (25.0)	9 (75.0)	12	7 (58.3)	5 (41.7)	0	0	11	8 (72.7)	3 (27.3)
Physician for CAM	13 (18.6)	12	11 (91.7)	1 (8.3)	12	6 (50.0)	6 (50.0)	12	10 (83.3)	1 (8.3)	1 (8.3)	0	11	4 (36.4)	7 (63.6)
Non-medical health provider (Heilpraktiker)	10 (14.3)	9	5 (55.6)	4 (44.4)	9	1 (11.1)	8 (88.9)	9	5 (55.6)	3 (33.3)	1 (11.1)	0	9	9 (100.0)	0
Osteopath	13 (18.6)	12	4 (33.3)	8 (66.7)	12	1 (8.3)	11 (91.7)	12	7 (58.3)	5 (41.7)	0	0	13	9 (69.2)	4 (30.8)
Chirotherapist	3 (4.3)	3	1 (33.3)	2 (66.7)	3	1 (33.3)	2 (66.7)	3	2 (66.7)	1 (33.3)	0	0	2	2 (100.0)	0
Other CAM provider^c^	14 (20.0)	—	—	—	—	—	—	—	—	—	—	—	—	—	—
Section 2: “Received CAM treatment”															
Homeopathy	6 (8.6)	5	3 (60.0)	2 (40.0)	5	5 (100.0)	0	5	4 (80.0)	1 (20.0)	0	0	6	4 (66.7)	2 (33.3)
Acupuncture	12 (17.1)	10	4 (40.0)	6 (60.0)	10	2 (20.0)	8 (80.0)	10	5 (50.0)	5 (50.0)	0	0	10	9 (90.0)	1 (10.0)
Herbal medicine	24 (34.3)	23	16 (69.6)	7 (30.4)	23	14 (60.9)	9 (39.1)	23	12 (52.2)	5 (21.7)	0	6 (26.1)	23	14 (60.9)	9 (39.1)
Manual therapy	20 (28.6)	19	9 (47.4)	10 (52.6)	20	5 (25.0)	15 (75.0)	20	13 (65.0)	7 (35.0)	0	0	18	13 (72.2)	5 (27.8)
Traditional Chinese Medicine	3 (4.3)	3	2 (66.7)	1 (33.3)	3	1 (33.3)	2 (66.7)	3	2 (66.7)	1 (33.3)	0	0	3	1 (33.3)	2 (66.7)
Other CAM treatments^c^	5 (7.1)	—	—	—	—	—	—	—	—	—	—	—	—	—	—
Section 3: “Herbal drugs and dietary supplements”															
Homeopathic remedies	20 (28.6)														
Traumeel	6 (8.6)	6	4 (66.7)	2 (33.3)	5	1 (20.0)	4 (80.0)	5	4 (80.0)	1 (20.0)	0	0	6	4 (66.7)	2 (33.3)
Nux vomica	4 (5.7)	4	2 (50.0)	2 (50.0)	4	2 (50.0)	2 (50.0)	4	3 (75.0)	1 (25.0)	0	0	4	2 (50.0)	2 (50.0)
Tissue salt (Schüssler)	3 (4.3)	3	1 (33.3)	2 (66.7)	3	0	3 (100.0)	3	0	3 (100.0)	0	0	3	3 (100.0)	0
Arnica	3 (4.3)	3	1 (33.3)	2 (66.7)	3	0	3 (100.0)	3	3 (100.0)	0	0	0	3	3 (100.0)	0
Herbs/Plant medicine	29 (41.4)														
Tumeric/Curcumin	9 (12.9)	9	8 (88.9)	1 (11.1)	9	3 (33.3)	6 (66.7)	9	4 (44.4)	5 (55.6)	0	0	9	2 (22.2)	7 (77.8)
Herbal teas	5 (7.1)	5	2 (40.0)	3 (60.0)	5	1 (20.0)	4 (80.0)	5	5 (100.0)	0	0	0	5	4 (80.0)	1 (20.0)
Mistletoe	3 (4.3)	3	3 (100.0)	0	3	0	3 (100.0)	3	0	1 (33.3)	0	2 (66.7)	3	2 (66.7)	1 (33.3)
Aromatherapy/essential oils	3 (4.3)	3	1 (33.3)	2 (66.7)	3	0	3 (100.0)	3	0	3 (100.0)	0	0	3	2 (66.7)	1 (33.3)
Sinupret	3 (4.3)	3	0	3 (100.0)	3	1 (33.3)	2 (66.7)	3	3 (100.0)	0	0	0	3	3 (100.0)	0
Iberogast	3 (4.3)	3	0	3 (100.0)	3	0	3 (100.0)	3	1 (33.3)	2 (66.7)	0	0	3	2 (66.7)	1 (33.3)
Indol-3-Carbinol	2 (2.9)	2	2 (100.0)	0	2	0	2 (100.0)	2	1 (50.0)	0	0	1 (50.0)	2	2 (100.0)	0
Vitamins/Minerals	60 (85.7)														
Vitamin D	40 (57.1)	40	33 (82.5)	7 (17.5)	40	16 (40.0)	24 (60.0)	39	32 (82.1)	5 (12.8)	0	2 (5.1)	37	20 (54.1)	17 (45.9)
Magnesium	21 (30.0)	21	10 (47.6)	11 (52.4)	21	6 (28.6)	15 (71.4)	21	15 (71.4)	3 (14.3)	0	3 (14.3)	21	13 (61.9)	8 (38.1)
Multinutritional supplement^d^	17 (24.3)	15	13 (86.7)	2 (13.3)	15	6 (40.0)	9 (60.0)	15	10 (66.7)	4 (26.7)	0	1 (6.7)	17	5 (29.4)	12 (70.6)
Vitamin B12	13 (18.6)	12	8 (66.7)	4 (33.3)	12	5 (41.7)	7 (58.3)	13	9 (69.2)	1 (7.7)	1 (7.7)	2 (15.4)	12	7 (58.3)	5 (41.7)
Selenium	12 (17.1)	12	9 (75.0)	3 (25.0)	12	4 (33.3)	8 (66.7)	12	9 (75.0)	2 (16.7)	0	1 (8.3)	12	8 (66.7)	4 (33.3)
Vitamin B-Complex	10 (14.3)	10	7 (70.0)	3 (30.0)	10	4 (40.0)	6 (60.0)	10	8 (80.0)	1 (10.0)	0	1 (10.0)	10	4 (40.0)	6 (60.0)
Other natural drugs	25 (35.7)														
Omega-3	13 (18.6)	13	8 (61.5)	5 (38.5)	13	6 (46.2)	7 (53.8)	13	10 (76.9)	1 (7.7)	0	2 (15.4)	12	5 (41.7)	7 (58.3)
Equinovo	4 (5.7)	4	4 (100.0)	0	4	1 (25.0)	3 (75.0)	4	4 (100.0)	0	0	0	4	2 (50.0)	2 (50.0)
Probiotics	3 (4.3)	3	1 (33.3)	2 (66.7)	3	0	3 (100.0)	3	3 (100.0)	0	0	0	3	1 (33.3)	2 (66.7)
Section 4: “Self-help practices”															
Meditation	29 (41.4)	28	27 (96.4)	1 (3.6)	29	21 (72.4)	8 (27.6)	29	22 (75.9)	7 (24.1)	0	0	24	13 (54.2)	11 (45.8)
Yoga	29 (41.4)	28	21 (75.0)	7 (25.0)	29	13 (44.8)	16 (55.2)	29	25 (86.2)	4 (13.8)	0	0	28	19 (67.9)	9 (32.1)
Qi Gong	15 (21.4)	15	10 (66.7)	5 (33.3)	15	8 (53.3)	7 (46.7)	15	10 (66.7)	5 (33.3)	0	0	12	5 (41.7)	7 (58.3)
Thai Chi	3 (4.3)	3	1 (33.3)	2 (66.7)	3	0	3 (100.0)	3	2 (66.7)	1 (33.3)	0	0	3	2 (66.7)	1 (33.3)
Relaxation techniques	38 (54.3)	36	30 (83.3)	6 (16.7)	37	24 (64.9)	13 (35.1)	36	30 (83.3)	6 (16.7)	0	0	32	18 (56.3)	14 (43.8)
Visualization	20 (28.6)	20	18 (90.0)	2 (10.0)	20	10 (50.0)	10 (50.0)	19	15 (78.9)	3 (15.8)	0	1 (5.3)	18	10 (55.6)	8 (44.4)
Praying for one’s own health	24 (34.3)	24	21 (87.5)	3 (12.5)	24	5 (20.8)	19 (79.2)	23	17 (73.9)	6 (26.1)	0	0	21	17 (81.0)	4 (19.0)
Paint/make music for own health	14 (20.0)	14	9 (64.3)	5 (35.7)	14	3 (21.4)	11 (78.6)	13	10 (76.9)	3 (23.1)	0	0	13	9 (69.2)	4 (30.8)
Other self-help practice^c^	26 (37.1)	—	—	—	—	—	—	—	—	—	—	—	—	—	—
Total	70 (100.0)	—	—	—	—	—	—	—	—	—	—	—	—	—	—

Abbreviations: n, number of patients; CAM, complementary and alternative medicine.

a Patients could select more than 1 CAM modality.

b Percentages in relation to the valid number of patients, who used the corresponding CAM modality.

c Number of patients who mentioned at least one other CAM modality (eg, CAM provider: physiotherapists; CAM-treatments: micronutrient-infusions; Self-help practices: physical activity, creativity, nature and music).

d All supplements with ≥3 substances.

Furthermore, vitamin D, relaxation techniques, and meditation were used the most in the context of cancer, and most participants became aware of relaxation techniques, meditation and vitamin D through the IO-GP. Vitamin D, relaxation techniques and yoga were rated as the most helpful CAM modalities and were also the most commonly mentioned modalities that had already been used before participating in the IO-GP.

### CAM Usage and Resilience, Physical QoL and Mental QoL

The effects of specific self-help practices on resilience, physical QoL and mental QoL are shown in Table 4. As a result, patients who practiced meditation and visualization within the last 12 months showed significantly greater mean values of resilience that did those who did not practice these modalities (meditation: *P* = .010, *d* = −.642 [medium effect size]; visualization: *P* = .003, *d* = −.805 [large effect size]). Furthermore, patients who reported yoga practices within the last 12 months had significantly greater physical QoL scores than did those who did not practice yoga (Mdn = 51.45; *R* = 39.22 vs Mdn = 42.36; *R* = 45.75; *P* = .003; *r* = .356 [medium effect size]).

**Table 4.** Effects of Specific Self-Help Practices on Resilience, Physical QoL, and Mental QoL.

Self-help practice	Resilience				Physical QoL (SF-P)				Mental QoL (SF-M)			
	n (%)	M ± SD	*P*-value*	Effect size (Cohen’s *d*)	n (%)	Mdn ± *R*	*P*-value**	Effect size (*r*)	n (%)	Mdn ± *R*	*P*-value**	Effect size (*r*)
Meditation												
No	41 (58.6)	65.78 ± 12.33	.**010**	−.642	40 (58.0)	44.00 ± 47.65	.236	.142	40 (58.0)	45.81 ± 38.63	.813	.029
Yes	29 (41.4)	72.66 ± 7.84			29 (42.0)	49.95 ± 37.32			29 (42.0)	45.63 ± 39.42		
Yoga												
No	41 (58.6)	66.80 ± 12.06	.105	−.399	40 (58.0)	42.36 ± 45.75	.**003**	.356	40 (58.0)	46.28 ± 38.63	.261	.135
Yes	29 (41.4)	71.21 ± 9.39			29 (42.0)	51.45 ± 39.22			29 (42.0)	43.54 ± 39.42		
Relaxation techniques												
No	32 (45.7)	68.56 ± 9.07	.964	−.011	32 (46.4)	42.45 ± 34.16	.056	.230	32 (46.4)	47.21 ± 37.53	.467	.088
Yes	38 (54.3)	68.68 ± 12.80			37 (53.6)	51.11 ± 47.65			37 (53.6)	45.17 ± 39.42		
Visualisation												
No	50 (71.4)	66.20 ± 11.81	.003	−.805	49 (71.0)	44.31 ± 47.65	.302	.124	49 (71.0)	45.76 ± 38.63	.606	.062
Yes	20 (28.6)	74.70 ± 6.26			20 (29.0)	50.33 ± 37.32			20 (29.0)	45.28 ± 35.27		
Praying for one’s own health												
No	46 (65.7)	67.96 ± 10.40	.490	−.175	45 (65.2)	45.86 ± 45.75	.533	.075	45 (65.2)	47.03 ± 41.93	.895	.016
Yes	24 (34.3)	69.92 ± 12.65			24 (34.8)	47.74 ± 30.73			24 (34.8)	45.00 ± 30.86		
Total	70 (100.0)	68.63 ± 11.17	—	—	69 (100.0)	46.83 ± 47.65	—	—	69 (100.0)	45.76 ± 41.93	—	—

Abbreviations: QoL, quality of life; n, number of patients; M, mean; SD, standard deviation; Mdn, median; R, range.

* Independent *t*-test (2-sided *P* value; bold *P*-values indicate significant differences; *P* < .05).

** Mann—Whitney-*U*-test (asymp. significance; bold *P*-values indicate significant differences; *P* < .05).

In addition, of the 29 patients who practiced meditation, those who practiced daily (n = 8) within the last 3 months exhibited significantly greater mean resilience scores than did those who practiced weekly (n = 15) (76.88 ± 7.97 vs 69.53 ± 7.47; *P* = .040, *d* = .961 [large effect size]). Among the 24 patients who prayed for their own health, those who prayed daily (n = 12) had significantly greater mean resilience scores than did patients who prayed weekly (n = 9) (74.92 ± 5.53 vs 63.11 ± 17.83; *P* = .042, *d* = .959 [large effect size]). Lastly, patients who practiced relaxation techniques daily (n = 10) had significantly lower mental QoL scores than did those who practiced relaxation techniques weekly (n = 19) (Mdn = 38.09; *R* = 25.54 vs Mdn = 47.28; *R* = 32.78; *P* = .019, *r* = .435 [medium effect size]).

### Safety Aspects

The survey revealed no medical risks associated with participation. Relating the safety of participating in the IO-GP, Savaş et al^25^ recorded no relevant side effects or adverse events related to the participation of the IO-GP. Reported side effects or safety aspects regarding the practice of specific CAM modalities were not surveyed in this study.

## Discussion

This study examined the long-term effects of a 10-week IO-GP on resilience and the use of CAM in 70 patients who completed the IO-GP 1 to 4.5 years ago. Study results show that resilience scores increased significantly ≥12 months after the IO-GP, compared to resilience scores before the IO-GP, and these changes had a medium effect size. Furthermore, the most frequently used CAM modalities after the IO-GP were vitamins/minerals, relaxation techniques, herbs and plant medicine, yoga and meditation. Practicing meditation and visualization was significantly associated with greater levels of resilience, while practicing yoga exhibited significantly greater levels of physical QoL.

The high proportion of female participants with breast cancer in this study underlines the heightened interest of women facing breast cancer in complementary treatment approaches and IO-GPs. This observation aligns with the findings of various other studies exploring predictors of CAM utilization during cancer disease, indicating that patients with breast cancer generally exhibit high levels of CAM use.^16,40–42^ Additionally, female gender, young age and a high education level were shown to be strong predictors of CAM usage after receiving a cancer diagnosis,^14^ especially for holistic methods from mind-body medicine approaches.^43^

Furthermore, the study population cohort exhibited predominantly health-conscious lifestyle behaviors, mostly aligning with the recommendations of DGE^30^ and the WHO^32,33^ concerning Mediterranean nutrition, avoiding smoking, having low-risk alcohol consumption and exercising regularly. However, there is room for improvement in vegetable and olive oil consumption. Meeting general health-related recommendations has been shown to play a major role in improving global health and general well-being,^44^ as well as cancer survivorship.^45^ In that regard, our IO-GP addresses the importance of health-promoting lifestyle modifications by schedule.^29^ A randomized controlled trial (RCT) by Ruiz-Vozmediano et al supported the idea, that multidisciplinary programs focusing on diet, exercise and mindfulness could strengthen patients’ ability to maintain a healthy lifestyle.^26^

Our primary study objective revealed that resilience levels increased significantly ≥12 months (1-4.5 years) after the IO-GP compared to resilience levels immediately before the program (baseline). In a previous study by Savaş et al,^25^ a slight increase in resilience was found for 52 patients immediately after the IO-GP (week 0: 63.50 ± 13.14; week 10: 66.15 ± 10.17); however, this difference was not statistically significant (*P* > .05). For the 44 patients in our study, the resilience scores continued to increase within 1 to 4.5 years post-group program, as we observed a shift from “low resilience” to “medium resilience.” Since there was no significant effect on the change in resilience levels of patients according to the subcategories “12 to 24 months,” “24 to 36 months,” and “>36 months,” further potential factors that influence resilience in patients with cancer and cancer survivors should be considered.

In that regard, existing literature indicates that distress, stigmatization and pain can negatively influence resilience in patients with cancer, while psychosocial resources, social support, a hopeful mindset and greater family members’ resilience levels are positively associated with patients’ resilience scores.^46–49^

Interventions from CAM have shown potential to enhance resilience in patients with cancer. A review by Ludolph et al^9^ found that resilience and posttraumatic growth increased notably with interventions such as positive psychology, supportive–expressive group therapy, behavioral therapy, and mindfulness, particularly with at least 12 therapeutic sessions. Our IO-GP consists of 10 sessions, so providing more sessions in the IO-GP schedule might represent a possible basis for improving the effectiveness of IO-GPs on resilience outcomes. Ludolph et al also observed lasting effects of supportive and complementary interventions on resilience and posttraumatic growth for up to 1 year post-intervention,^9^ which is also emphasized in other studies on IO-GPs, demonstrating sustained improvements in QoL^23^ and healthy lifestyle behavior^26^ over a 6-month follow-up period. In conclusion, greater levels of resilience and QoL seem to be associated with maintaining certain CAM modalities, especially mindfulness and positive psychology. This is in line with our study results, which indicate that patients practicing “meditation” and “visualization” had significantly greater resilience scores compared to non-practitioners, and practicing “yoga” was significantly associated with greater physical QoL scores.

A meta-analysis by Cillessen et al^50^ on mindfulness-based interventions for psychological and physical health indicated that meditation interventions have the potential to reduce psychological distress, fatigue, sleep disturbance, pain, anxiety, and depression. In turn, the absence of these components promotes greater resilience and QoL.^9^ Mindfulness-based interventions from mind-body medicine, such as relaxation, meditation, and yoga, are important elements of our IO-GP, and our findings indicate that a considerable number of patients with cancer still use these CAM approaches. To compare these findings with the literature, the researcher team of the international I-CAM-Q questionnaire^36^ published a study about the prevalence of CAM in patients with cancer in Norway. Kristoffersen et al^17^ reported that 79% (n = 346) of the study population had used some kind of CAM (mean 3.8 modalities, range 1-17). With respect to self-help practices in that study, some patients with cancer reported practicing relaxation techniques (48.7%, n = 213), meditation/mindfulness (29.1%, n = 127) and yoga (27.9%, n = 122). Compared with our study population, the relative percentages of patients who used relaxation techniques (54.3%, n = 38), meditation and yoga (both 41.4%, n = 29) were greater. This evidence, combined with the fact that a substantial proportion of patients within our study cohort reported becoming aware of mindfulness-based practices through participating in the IO-GP—specifically for relaxation techniques (64.9%), meditation (72.4%), and yoga (44.8%)—provides preliminary indications that the IO-GP may effectively promote the utilization of CAM. However, it is important to emphasize that the relative number of patients with breast cancer was greater in our study than in that of Kristoffersen et al (83.1% vs 39.1%).

Patients with breast cancer have been reported to exhibit a greater utilization of CAM in existing literature, which may contribute to the findings of our study.^16,40–42^ The fact that mostly female patients with breast cancer made up our study population is likely to have an influence on patients’ compliance and study outcomes regarding the continuation of CAM interventions after integrative oncological approaches. Consequently, our study population may have limitations in representing the broader population of patients with cancer.

Another relevant fact regarding the representativeness of our study cohort is that a certain number of patients who participated in the IO-GP had passed away at the time of the survey, could not be reached, refused to take part in the study or did not fill out the questionnaire. This resulted in a selection bias that limits the representativeness of our study population for investigating the long-term effects of the IO-GP on resilience and the use of CAM. Nevertheless, the study results highlight the long-term effects of participating in the IO-GP relating to the sustainable use of certain CAM modalities, particularly for female patients with breast cancer.

Furthermore, the literature points out that CAM is particularly utilized by women with higher educational status and income,^14,16,40,42^ which is consistent with our study findings. However, the maintenance of CAM after the IO-GP can be associated with further costs. Wode et al^14^ reported that more than half of the patients with cancer in their study spent ≤50 € monthly on CAM, with >90% reporting that it was worth the money. Reimbursement for CAM in Germany is only partially covered by public health insurances, leading to the conflict that patients with a lower socioeconomic status may have limited access to certain CAM modalities. To partially address this issue, our department provides free access to the IO-GP and additional mindfulness courses for patients with cancer through support from foundations.^28^ Additionally, several public health insurance companies in Germany cover the costs for further integrative oncological programs.^23^

In addition, the exact reasons for the use of specific CAM modalities were not determined in our study. Studies by Kristoffersen et al show that 43.0% of the study population reported using some type of CAM to alleviate long-term symptoms such as fatigue, sleep disorders, and hot flashes; mostly through self-help practices (26.0%), including relaxation, meditation, and yoga.^40^ Most patients used these self-help practices to increase QoL, for coping, relaxation or well-being (96.0%), but also to strengthen the body and immune system (46.2%) or to treat side effects or late effects of cancer and its treatment (39.9%).^17^

Furthermore, a small percentage (6.3%) of patients in a study by Kristoffersen et al also reported experiencing adverse effects associated with certain CAM modalities, including relaxation techniques, meditation/mindfulness, yoga, and visualization.^17^ However, these adverse events were mostly mild or moderate, indicating low rates of adverse effects overall. Savaş et al^25^ found that patients participating in our IO-GP did not report relevant side effects or adverse events. Some patients experienced difficulties during the program, such as recalling childhood memories or empathizing with others’ problems.

Moreover, a significant proportion of our patients mentioned the use of vitamin and mineral supplements (85.7%). Notably, vitamin D emerged prominently, as stated by 57.1% of the study cohort, and was mainly used in connection with the cancer diagnosis. In addition, 40.0% of the study participants became aware of vitamin D through the IO-GP, which illustrates that the information provided about nutritional supplements in our IO-GP can influence adequate vitamin D supplementation even after its completion. In that regard, prior study results suggest that adequate vitamin D levels are associated with the prevention and reduction of susceptibility to breast cancer and cancer recurrence.^51,52^

The high satisfaction levels reported with self-help practices, particularly yoga and relaxation techniques, emphasize their positive impact on patients with cancer. Prior reviews, as well as practical medical guidelines, have indicated that yoga interventions are most often recommended to improve QoL in patients with breast cancer.^18,19,53^ However, in our study, daily practice of relaxation techniques was significantly associated with lower mental QoL, which is in contrast with the high satisfaction rate of relaxation techniques and prior study results, showing improvements in overall QoL with MBRS^24,54^ and muscle relaxation techniques.^55,56^ Furthermore, it is conceivable that patients who had used relaxation techniques daily might have experienced high levels of stress, requiring daily intervention through relaxation practices. In addition, the I-CAM-G questionnaire does not explicitly survey the specific type of relaxation technique, which leads to limitations in determining the effectiveness of the practiced techniques.

Last, it is remarkable that more than half of the participants had already used self-help practices before participating in the IO-GP. Whether these patients had already practiced those modalities regularly, or only sporadically, cannot be determined exactly with our questionnaire. However, it might be possible that the IO-GP has encouraged already practicing patients to actively practice these self-help techniques again, whereas newcomers to mindfulness-based practices might be supported in implementing these techniques in their life. Consequently, as the IO-GP equips patients suffering from cancer with knowledge and skills relating to CAM and healthy lifestyle behaviors, one can assume that IO-GPs hold the potential to empower cancer survivors to continue using CAM practices for ongoing health, QoL and resilience.

Overall, our findings provide valuable clinical insights into the long-term effects of our IO-GP on resilience and CAM usage behavior, with significant increases in resilience levels observed ≥12 months after the IO-GP. Practicing certain self-help strategies may play a crucial role in promoting resilience and enhancing QoL in patients with cancer. We recommend empowering patients with cancer to participate in IO-GPs and continue specific self-help strategies to improve their resilience and overall well-being.

## Limitations

This study has certain limitations. Examination of longitudinal changes in resilience values was limited due to the small amount of available baseline data and the small number of patients included in the subgroup analyses. Moreover, there was no control group that could be compared with our study population. Additionally, the COVID-19 pandemic restricted certain group program organizations in the past, so patients affected could not have equal experiences as patients not affected by COVID-19 restrictions. Data on QoL (SF-12) and CAM usage (I-CAM-G) were collected during the survey for the first time, so baseline data from the time before the IO-GP did not exist, precluding a potential comparison. Furthermore, the reasons for patients’ utilization of specific CAM modalities were not documented. Medical risks related to this study and participation in the IO-GP were not surveyed/monitored. As our study cohort mostly consisted of female patients with breast cancer, our study results may have limitations in representing the broader population of patients with cancer. Furthermore, a certain number of patients, who took part in the IO-GP, had passed away at the time of the survey, could not be reached, refused to take part in the study or did not fill out the questionnaire.

## Conclusion

A significant increase in resilience was observed ≥12 months after completing the IO-GP (medium resilience) compared to before participation (low resilience), while the time elapsed since completing the IO-GP ranged from 1 to 4.5 years and showed no significant impact on the observed change in resilience. The most frequently reported CAM modalities included vitamins/minerals, relaxation techniques, herbs and plant medicine, meditation, and yoga. Significant effects on resilience were found for meditation and visualization, while yoga practice was significantly associated with greater physical QoL. In conclusion, a general recommendation can be given for maintaining a regular practice of mindfulness-based techniques, as well as maintaining a health-conscious lifestyle during and after cancer, as these recommendations represent important foundations for promoting general well-being and health after cancer. In this context, this recommendation can be made specifically for female patients with gynecological cancers, especially breast cancer. To support this goal in a cancer therapy setting, broad and eased access to IO-GPs is desirable. In addition, further research is needed to examine the long-term effects of IO-GPs on resilience and the utilization of CAM, with high-quality safety monitoring to address potential adverse effects and medical risks.
